# Viral Suppression Among People Living with HIV in Tajikistan: A Nationwide Analysis

**DOI:** 10.3390/v18030348

**Published:** 2026-03-12

**Authors:** Kamiar Alaei, Brian Kwan, Christopher P. Lounsbery, Jamoliddin Abdullozoda, Salomudin J. Yusufi, Patricia Cortez, Mannat Tiwana, Julie Nguyen, Hamid R. Torabzadeh, Arash Alaei

**Affiliations:** 1 Department of Health Science, College of Health and Human Services, California State University Long Beach, Long Beach, CA 90840, USAarash.alaei@csulb.edu (A.A.); 2 Center for Global Health, College of Health and Human Services, California State University Long Beach, Long Beach, CA 90840, USA; 3 Ministry of Health and Social Protection of the Population, Dushanbe 734025, Tajikistan; 4 Faculty of Surgery, Avicenna Tajik State Medical University, Dushanbe 734000, Tajikistan; 5 Faculty of Pharmacology, Avicenna Tajik State Medical University, Dushanbe 734000, Tajikistan; 6 School of Public Health, Brown University, Providence, RI 02912, USA

**Keywords:** viral suppression, Tajikistan, Central Asia, UNAIDS, rural areas, gender disparity, age, regional disparity

## Abstract

Viral suppression is a cornerstone of HIV management, essential for improving health outcomes and preventing transmission. However, varying definitions of suppression, ranging from ≤1000 copies/mL (controlled) to ≤200 (clinically suppressed) and ≤50 (untransmittable), complicate the assessment of progress toward global UNAIDS 95–95–95 goals. Our study evaluated progress in achieving viral suppression among people living with HIV (PLHIV) in Tajikistan between 2010 and 2024 using cross-sectional data from the Ministry of Health and Social Protection of Population registry. Viral load was measured using real-time PCR, and suppression was assessed across three thresholds (≤1000, ≤200, ≤50 copies/mL). We examined associations between viral suppression and demographic factors using Chi-square tests and logistic regression models. Across all thresholds, suppression rates remained below the UNAIDS 95-95-95 target goals. At the ≤50 copies/mL threshold, 77% of males and 83% of females achieved suppression, with males demonstrating lower odds of achieving viral suppression. Regional disparities were evident, with Khatlon and Sughd showing the lowest viral suppression rate (72.2% and 76.8%, respectively) and lower odds of achieving viral suppression compared to Dushanbe. Urban–rural differences were also observed (78.3% vs. 81.1%), though odds ratios using logistic regression models were not significant. Findings highlight persistent demographic and regional disparities, underscoring the need for targeted interventions to achieve equitable viral suppression in Tajikistan. Our findings also highlight associations and do not imply causal inference. In addition, authors acknowledge that interpretation of viral suppression outcomes is limited by the absence of data on treatment regimens, duration, adherence, CD4 counts, and behavioral factors.

## 1. Introduction

Given that viral suppression is a critical component in the management of HIV/AIDS, achieving this goal is essential not only for improving individual health outcomes but also for preventing the transmission of HIV within communities [1,2]. According to the World Health Organization (WHO), viral load is considered suppressed at ≤1000 copies/mL, with values between 200 and 1000 copies/mL representing low-level suppression and <200 copies/mL considered undetectable [1,3]. However, other definitions are proposed for undetectable with values as low as having <50 copies/mL [4]. These different definitions of viral suppression have set the precedent in “treatment as prevention” initiatives, capitalizing on the fact that undetectable viral loads are untransmittable (U=U) [5]. Antiretroviral therapy (ART) plays a pivotal role in this process, effectively slowing disease progression, preserving immune function, and significantly enhancing the quality of life for people living with HIV (PLHIV) [6,7]. Adherence to ART is especially important for achieving viral suppression and undetectable viral loads, since lack of adherence can lead to adverse effects, including drug resistance mutations or congenital transmission [8]. It is important to note that maintaining an undetectable viral load requires consistent adherence to ART, as undetectability must be continuously sustained through ongoing treatment. Poor adherence can lead to fluctuations in viral suppression, particularly affecting undetectable levels, and may increase the risk of HIV transmission [9].

As global health initiatives strive to meet UNAIDS’ 95-95-95 targets by 2030 [10], there has been greater importance placed on achieving high rates of viral suppression. As of 2024, global estimates narrowly missed the UNAIDS’ first 95 target, with 87% of PLHIV aware of their status [11]. Of those, 89% were receiving treatment, and among those on treatment, 94% were virally suppressed (≤1000 copies/mL) [11]. A key area of focus in the global response to the HIV epidemic is Central Asia, particularly Tajikistan. Although data from the early 2000s indicated relatively low HIV prevalence across Tajikistan, the country experienced one of the fastest rates of growth in new HIV infections globally [12]. HIV transmission, while initially facilitated through injectable drug use, has expanded to a number of routes initiated by key populations including labor migrants, sex workers, and men who have sex with men. Following the dissolution of the Soviet Union, economic constraints significantly weakened Tajikistan’s public health infrastructure, exposing chronic funding shortfalls within the regional healthcare system [12]. These limitations restricted access to HIV testing, treatment, and care, contributing to delayed diagnoses and suboptimal early responses to the epidemic [12]. Although few studies highlight HIV care cascade data in Tajikistan, recent PEPFAR reports demonstrate UNAIDS 95-95-95 targets reaching 69%, 92%, and 94%, respectively [13]. While these findings showcase progress towards meeting the UNAIDS targets, current data lacks threshold definitions, making it difficult to translate global progress. By applying three commonly used suppression definitions to the nationwide registry data, this study provides a clearer and more nuanced assessment of Tajikistan’s progress toward the UNAIDS 95–95–95 targets. Additionally, we aim to further investigate whether these recent PEPFAR reports align with nationwide data from the Tajikistan Ministry of Health and Social Protection of Population (MOHSPP) and support current U=U initiatives. The objective of this paper is to examine the viral suppression progress PLHIV in Tajikistan.

## 2. Materials and Methods

**Figure 1:**
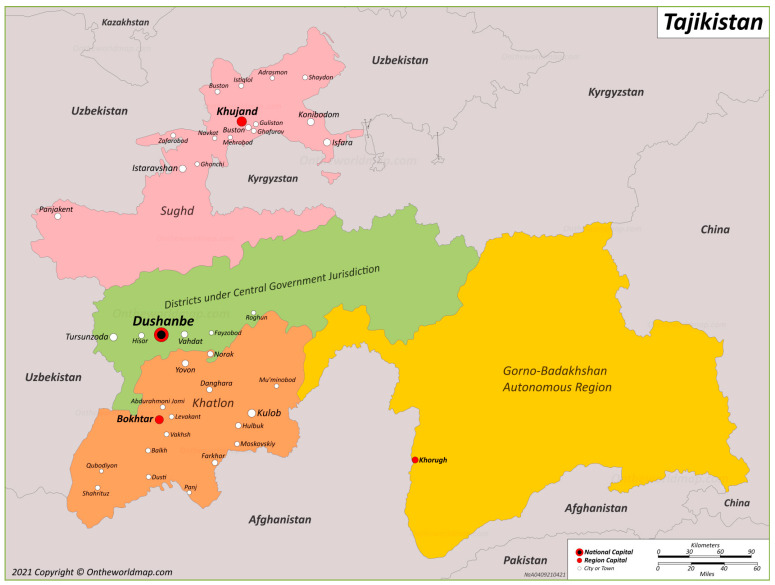
Administrative regions (oblasts) of Tajikistan and major cities. Adapted from OnTheWorldMap (2021) [15].

Our study examines the nationwide, cross-sectional data of (N = 12,487) individuals living with HIV, with their diagnosis of HIV occuring anytime between the years of 2010 and 2024, from the national registry system managed by the Tajikistan MOHSPP. Tajikistan is divided into four main Oblasts (regions/provinces), which include Sughd, Khatlon, the Gorono–Badakhsan Autonomous Region (GBAO) and a region surrounding the capital city of Dushanbe, the Districts of Republican Subordination (DRS) (Figure 1). Data was collected from individuals residing in each of these Oblasts as well as Dushanbe, comprising five regions in Tajikistan.

To gauge viral suppression levels for PLHIV, viral load count measurement was conducted using the real-time PCR method by Rotor-Gene Q and GeneXpert, manufactured by QIAGEN and Cepheid, respectively. Viral load testing was available in all districts of Tajikistan, and all testing services were free for our N = 12,487 PLHIV. The primary outcome measured the latest viral load measurement at least six months after ART initiation, for all Tajik PLHIV. Current antiretroviral treatment regimens in Tajikistan are based on and informed by WHO recommendations, including the preferred TDF/3TC/DTG regimen and alternative TDF/3TC/EFV regimen. HIV viral suppression was assessed by calculating the proportion of PLHIV with viral load in the following three thresholds: low-level suppression (≤1000 copies/mL), moderate suppression (detected but ≤200 copies/mL), and undetectable viral suppression (no detectable virus, <50 copies/mL).

Proportions of HIV viral load suppression were calculated to evaluate the effectiveness of treatment and care and illustrate differences in suppression status between the three thresholds. We investigated HIV viral suppression among demographics: age group (<20 y, 20–40 y, 41–59 y, >60 y), gender (Female, Male), region of residence (Dushanbe, DRS, GBAO, Khatlon, Sughd), and area of residence (Urban, Rural).

We conducted chi-square tests of independence to test for significant associations between viral suppression status (threshold achieved vs. not achieved) and demographics. Logistic regression models were developed to examine the direction and magnitude of associations between demographics and the likelihood of achieving viral suppression at the three defined thresholds, i.e., odds ratios (ORs), 95% confidence intervals (CIs). Multiple predictors were considered in the logistic regression models to allow for examination of any changes in associations and the significance between predictors and outcomes. Our focus is on the main effects of the predictors of our models; interaction terms were not included to preserve model parsimony and interpretability. *p*-values were examined for statistical significance at the 5% level. Individuals with complete data on the outcome and predictors were used to train our logistic regression models. Multicollinearity for our models was assessed using variance inflation factor (VIF), Cook’s Distance and DFBetas were examined to gauge model diagnostics, and Hosmer–Lemeshow (H-L)’s test was used to assess the goodness-of-fit for our models. Our statistical models did not experience any multicollinearity or challenges with model diagnostics and the models were all a good fit and well-calibrated (all H-L *p* > 0.05). All statistical analysis was conducted using the R programming language [14].

## 3. Results

### 3.1. Demographic Characteristics

**Figure 2:**
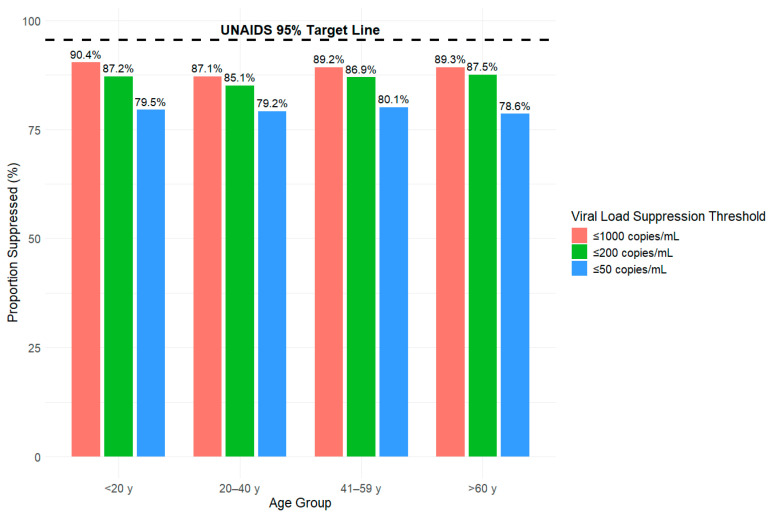
Proportion of HIV viral load suppression by age group (Years) and threshold (copies/mL) from 2010 to 2024 in Tajikistan.

**Figure 3:**
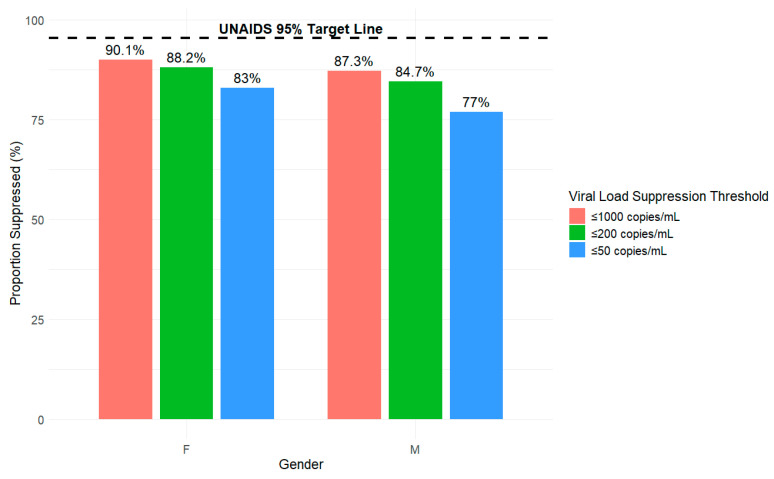
Proportion of HIV viral load suppression by gender (male or female) and threshold (copies/mL) from 2010 to 2024 in Tajikistan.

**Figure 4:**
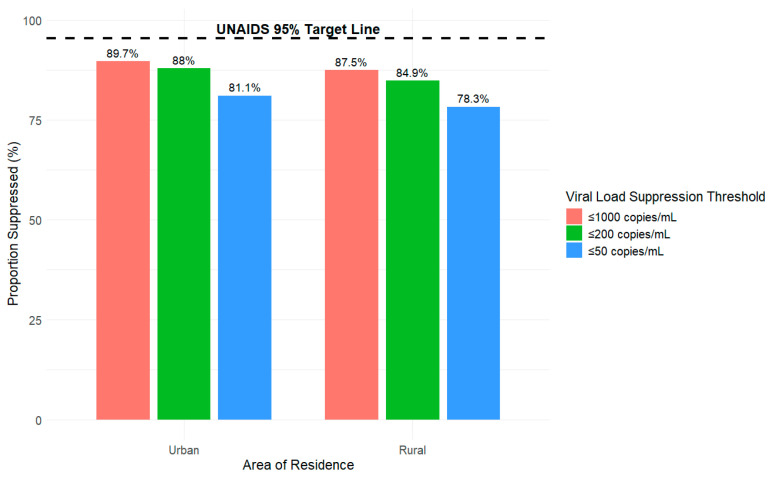
Proportion of HIV viral load suppression by area of residence (urban or rural) and threshold (copies/mL) from 2010 to 2024 in Tajikistan.

In Table 1, we present descriptive statistics for demographic characteristics of our overall study sample of HIV cases in Tajikistan from 2010 to 2024 (N = 12,487), stratified by viral suppression status (HIV-RNA≤ and >1000 copies/mL). Our overall study sample consists of approximately 40% of HIV cases of age 20–40 years and a similar proportion of HIV cases of age 41–59 years. The majority of HIV cases were male (53%) and were in rural areas of residence (53.2%). Dushanbe, Khalton, and Sughd each make up greater than 20% of HIV cases. All demographic characteristics were significantly associated with viral suppression status (all *p* < 0.05). The relationships between these demographics across different viral suppression thresholds, ≤200 and ≤50 copies/mL, were also examined in the follow-up Figure 1, Figure 2, Figure 3 and Figure 4 and Table 2.

In Figure 1, Figure 2, Figure 3 and Figure 4, we present the proportion (%) of HIV cases in Tajikistan who achieved viral suppression at the ≤1000, ≤200, and ≤50 copies/mL thresholds, stratified by demographic characteristics. Across the three defined viral suppression thresholds, rates of viral suppression varied slightly by groups within demographics and consistently fell below the UNAIDS global target of 95%.

**Table 1:** Demographic characteristics of study sample of N = 12,487 individuals (overall) living with HIV in Tajikistan, stratified by viral load HIV-RNA ≤ 1000 copies/mL (N = 10,202) and >1000 copies/mL (N = 1332). *p*-values obtained from Chi-square tests of independence between demographic characteristics and viral suppression status (≤ or >1000 copies/mL).

Variable	Overall	People with HIV-RNA ≤1000 Copies/mL	People with HIV-RNA >1000 Copies/mL	*p*-Value
Age Group (y)				0.001
<20	1214 (9.7)	1097 (9.8)	117 (5.1)	
20–40	4971 (39.8)	4311 (38.6)	636 (27.8)	
41–59	4943 (39.6)	4385 (39.3)	529 (23.1)	
>60	459 (3.7)	408 (3.7)	49 (2.1)	
Gender				<0.001
Male	6612 (53.0)	5735 (51.4)	838 (36.7)	
Female	4975 (39.8)	4466 (40)	493 (21.6)	
Region of Residence				<0.001
Dushanbe	2634 (21.1)	207 (9.1)	2424 (21.7)	
Districts of Republican Subordination (DRS)	2350 (18.8)	159 (7)	2181 (19.6)	
Gorno–Badakhshan Autonomous Province (GBAO)	440 (3.5)	37 (1.6)	401 (3.6)	
Khatlon	3553 (28.5)	643 (28.1)	2875 (25.8)	
Sughd	2591 (20.7)	281 (12.3)	2305 (20.7)	
Area of Residence				<0.001
Urban	4770 (38.2)	489 (21.4)	4268 (38.3)	
Rural	6640 (53.2)	823 (36)	5777 (51.8)	

### 3.2. Viral Suppression Disaggregated by Age

In Figure 2, at the ≤1000 copies/mL threshold, 90.4% of HIV cases under 20 y achieved viral suppression compared to 87.1% of those aged 20–40 y, 89.2% of those aged 41–59 y and 89.3% of those aged over 60 y. At the ≤200 copies/mL threshold, we saw suppression rates decrease slightly with 87.2% for those under 20 y, 85.1% for those aged 20–40 y, 86.9% for those aged 41–59 y, and 87.5% for those aged over 60 y. At the strictest threshold of ≤50 copies/mL, suppression rates decreased further across all age groups, with 79.5% among those under 20 y, 79.2% for those aged 20–40 y, 80.1% for those aged 41–59 y, and 78.6% for those aged over 60 y. 

### 3.3. Viral Suppression Disaggregated by Gender

In Figure 3, viral suppression rates across all three thresholds (≤1000, ≤200, and ≤50 copies/mL) fell below the UNAIDS 95% target, with females having overall better viral suppression outcomes compared to males. At the ≤1000 copies/mL threshold, 90.1% of females achieved viral suppression compared to 87.3% of males. At the ≤200 threshold, 88.2% of females achieved viral suppression compared to 84.7% of males. Despite the decreasing overall suppression rates at stricter thresholds, females maintained higher viral suppression rates compared to males across all three thresholds. At the strictest threshold of ≤50 copies/mL, 83% of females achieved viral suppression compared to 77% of males. Gender gap trends increased from 2.8% for viral suppression ≤ 1000 copies/mL (90.1–87.3%) to 6% for viral suppression ≤ 50 copies/mL (83–77%).

### 3.4. Viral Suppression Disaggregated by Area of Residence

In Figure 4, viral suppression rates differed between urban and rural areas across all three thresholds (≤1000, ≤200, and ≤50 copies/mL) but remained below the UNAIDS global target of 95%. At the ≤1000 copies/mL threshold, 87.5% of individuals in rural areas achieved viral suppression compared to 89.7% in urban areas. At the ≤200 copies/mL threshold, rates declined slightly, with 84.9% of rural areas achieving viral suppression compared to 88% in urban areas. At the strictest threshold of ≤50 copies/mL, suppression rates further declined to 78.3% in rural areas compared to 81.1% in urban areas. The area of residence gap trends increased from 2.2% for viral suppression ≤ 1000 copies/mL (89.7–87.5%) to 2.8% for viral suppression ≤ 50 copies/mL (81.1–78.3%). Across all thresholds, viral suppression rates were consistently lower in rural areas compared to urban areas.

### 3.5. Viral Suppression Disaggregated by Region of Residence

**Figure 5:**
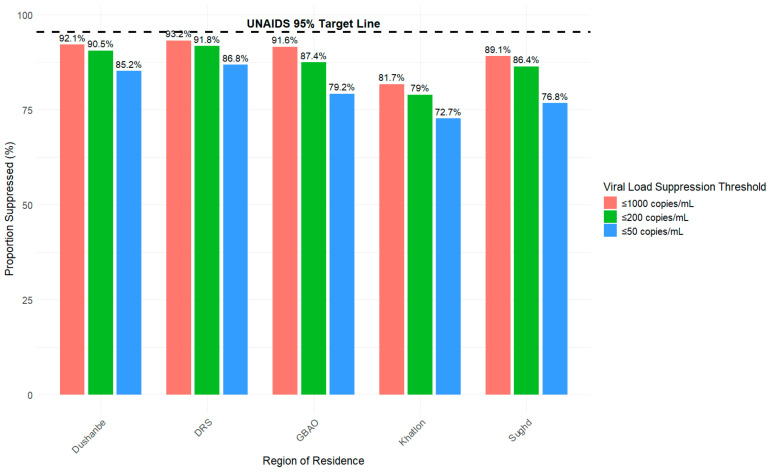
Proportion of HIV viral load suppression by region of residence (Dushanbe, DRS, GBAO, Khatlon, Sughd) and threshold (copies/mL) from 2010 to 2024 in Tajikistan.

In Figure 5, regional differences in viral suppression were likewise observed at each of the three thresholds; however, no region achieved the UNAIDS global target of 95%. At the ≤1000 copies/mL threshold, 93.2% of HIV cases in the Districts of Republican Subordination (DRS) region achieved viral suppression compared to 92.1% in the capital of Dushanbe, 91.6% in the Gorno–Badakhshan Autonomous Province (GBAO), 89.1% in Sughd; there was a noticeable drop to 81.7% in Khatlon. At the ≤200 copies/mL threshold, suppression rates decreased slightly for all regions with 91.8% achieving viral suppression in the DRS region compared to 90.5% in Dushanbe, 87.4% in GBAO, 86.4% in Sughd, and dropping to below 80% in Khatlon (79%). At the strictest threshold of ≤50 copies/mL, suppression rates declined further across all regions with 86.8% achieving viral suppression in DRS, 85.2% in Dushanbe, and multiple regions dropping below 80% (GBAO: 79.2%, Sughd: 76.8%, Khatlon: 72.7%). Across all thresholds, Khatlon experienced the lowest suppression rates among the five regions. Region of residence trends highlight progressive disparities between Khatlon and Sughd, and other regions. For instance, the viral suppression disparity between Khatlon and Dushanbe increased from 10.4% at ≤1000 copies/mL threshold to 12.5% for viral suppression at ≤50 copies/mL threshold. Additionally, the viral suppression disparity between Sughd and Dushanbe increased from 3% at ≤1000 copies/mL threshold to 8.4% for viral suppression at ≤50 copies/mL threshold.

### 3.6. Multiple Logistic Regression Results for Viral Suppression

In Table 2, we developed three multivariable logistic regression models (Models 1, 2, 3) to examine the associations between the demographic variables—age group, gender, area of residence, and region of residence—for each of the three binary outcomes of whether viral suppression was achieved or not at the three defined thresholds, ≤1000, ≤200, and ≤50 copies/mL. In Model 1, we see that the odds of achieving viral suppression at ≤1000 copies/mL were significantly increased for <20 y and 41–59 y compared to that of 20–40 y. Males had a 28% significantly decreased odds compared to that of females in achieving viral suppression at ≤1000 copies/mL (95% CI: 0.64, 0.82). HIV cases from Khatlon and Sughd had significantly decreased odds in achieving viral suppression at ≤1000 copies/mL compared to that of Dushanbe (the capital city) with Khatlon having 64% decreased odds (95% CI: 0.3, 0.44) and Sughd having 33% decreased odds (95% CI: 0.55, 0.83). In Model 2, the odds of achieving viral suppression at ≤200 copies/mL were significantly increased for 41–59 y compared to that of 20–40 y, which differs from the associations between age and ≤1000 copies/mL from Model 1. Much like Model 1, males had significantly decreased odds in achieving viral suppression compared to that of females, and HIV cases from Khatlon and Sughd had significantly decreased odds compared to that of Dushanbe. In Model 3, there was not a significant association between age and achieving viral suppression at ≤50 copies/mL, which differs from Models 1 and 2. Consistent with Models 1 and 2, males had significantly decreased odds in achieving viral suppression compared to that of females. Uniquely for Model 3, beyond Khatlon and Sughd, HIV cases from GBAO had significantly decreased odds of 36% (95% CI: 0.49, 0.83) in achieving viral suppression at ≤50 copies/mL compared to those in Dushanbe. Overall, gender and region of residence displayed consistent significant associations with all three thresholds accounting for the other demographics, with the statistical significance of the age groups varying by thresholds.

**Table 2:** Multivariable logistic regression models (multiple predictors) with binary outcome for achieving the viral suppression threshold (i.e., ≤1000, ≤200, and ≤50 copies/mL). Each model contains all demographic characteristics (i.e., age group, gender, area of residence, region of residence) as predictors. OR = odds ratio; CI = confidence interval; Ref. = reference group.

	Model 1: ≤1000 Copies/mL		Model 2: ≤200 Copies/mL		Model 3: ≤50 Copies/mL	
	OR (95% CI)	*p*-Value	OR (95% CI)	*p*-Value	OR (95% CI)	*p*-Value
**Age Group**						
<20 y	1.28 (1.04, 1.59)	0.023	1.09 (0.90, 1.32)	0.381	0.94 (0.8, 1.1)	0.423
20–40 y (Ref.)	—	—	—	—	—	—
41–59 y	1.22 (1.07, 1.38)	0.002	1.17 (1.04, 1.31)	0.009	1.09 (0.98, 1.2)	0.101
>60 y	1.16 (0.85, 1.6)	0.362	1.18 (0.89, 1.6)	0.26	0.96 (0.76, 1.23)	0.761
**Gender**						
Female (Ref.)	—	—	—	—	—	—
Male	0.72 (0.64, 0.82)	<0.001	0.71 (0.64, 0.8)	<0.001	0.66 (0.6, 0.73)	<0.001
**Area of Residence**						
Urban (Ref.)	—	—	—	—	—	—
Rural	1.06 (0.92, 1.22)	0.403	0.98 (0.86, 1.12)	0.794	1.06 (0.94, 1.18)	0.345
**Region of Residence**						
Dushanbe (Ref.)	—	—	—	—	—	—
DRS	1.1 (0.87, 1.41)	0.426	1.16 (0.93, 1.45)	0.186	1.07 (0.89, 1.29)	0.445
GBAO	0.90 (0.62, 1.33)	0.583	0.72 (0.53, 1.01)	0.052	0.64 (0.49, 0.83)	<0.001
Khatlon	0.36 (0.3, 0.44)	<0.001	0.39 (0.33, 0.47)	<0.001	0.43 (0.37, 0.5)	<0.001
Sughd	0.67 (0.55, 0.83)	<0.001	0.66 (0.55, 0.8)	<0.001	0.54 (0.46, 0.64)	<0.001

## 4. Discussion

While international-level data indicate significant progress toward the UNAIDS third 95 target, a more detailed analysis reveals significant disparities that must be addressed. Tajikistan has made notable advances, with approximately 94% of PLHIV on ART achieving viral suppression at the ≤1000 copies/mL threshold [13]. Our results indicate that while suppression rates at the higher threshold of ≤1000 copies/mL were relatively high and approached UNAIDS targets, lower thresholds at ≤200 and ≤50 copies/mL propose potential gaps in care and health-compromising behavior. In addition, our results demonstrate disparities in achieving viral suppression by variables such as age, gender, region of residence, and area of residence. 

Age played a role in viral suppression outcomes. Younger populations under 20 and individuals aged 41–59 showed better viral suppression compared to those aged 20–40, highlighting the need for targeted interventions for young adults. This may be linked to factors such as life stage transitions, migrant employment, engagement in sex work, injectable drugs, or challenges associated with maintaining long-term care among younger adults [16,17]. Furthermore, studies from other low-income countries have demonstrated that the stigma surrounding the disease and a lack of familial support affect health care-seeking behaviors and adherence to ART among young adults, which may affect viral suppression [18,19,20]. While age may influence the likelihood of achieving broader viral suppression targets such as HIV RNA < 1000 copies/mL, its impact appears diminished when considering more stringent thresholds like < 50 copies/mL. At the <50 copies/mL threshold, all age groups exhibited approximately 80% viral load suppression. Explanations for this trend in data may be related to issues with adherence to ART. This comes with the understanding that reaching viral suppression for this threshold is dependent on continued ART use [4].

Viral suppression rates were consistently higher among females compared to males at all thresholds, with data highlighting increasing gaps to viral suppression at stricter thresholds. This may reflect lower health-seeking behaviors among men, structural barriers, or a lack of male-targeted engagement strategies within HIV programs. The observed gender disparity may also reflect the influence of male-dominated key populations, including men who have sex with men (MSM) and migrant workers. Both groups experience unique structural and psychosocial barriers that may impact viral suppression. The majority of migrant workers from Tajikistan are male, often traveling to Russia for short-term work, seeking better economic opportunities [21]. Recent studies among Tajik migrant workers have reported low engagement in HIV care and reduced health-seeking behaviors, often driven by stigma and fears that disclosure of HIV status could jeopardize employment opportunities [21,22]. Additional literature indicates that this population frequently has limited knowledge of HIV transmission and personal status, further delaying initiation of ART and reducing the likelihood of achieving viral suppression [22]. Similarly, MSM often avoid HIV services in countries culturally comparable to Tajikistan due to stigma and harassment within healthcare settings, where discrimination based on sexual orientation deters disclosure and sustained engagement in treatment, which may have effects on viral suppression [23,24]. Additionally, this population is prone to high transmission of HIV and was responsible for most new infections in Central Asia in the early 2010s [25]. The lack of engagement in care and high transmission rates among MSM may likely play a role in the lack of viral suppression among men in Tajikistan. On the other hand, our findings indicate progress in women’s access to HIV care, relative to earlier reports, which found that cultural norms limited partnered women’s ability to seek care independently, contributing to gender disparities and challenges in monitoring female patients. This improvement suggests that previously observed gender gaps in HIV care may be narrowing [26]. Consistent with this, our previous study found earlier detection of HIV among females (CD4 ≥ 350 cells/μL) compared to males [27], a factor that may also enhance ART success and facilitate viral suppression.

Rural residents overall had slightly lower viral suppression rates compared to their urban counterparts, highlighting disparities between areas of residence. Other studies of rural communities have demonstrated factors such as long distances to clinics, scarcity of trained healthcare providers, loss to follow-up, and stigma as possible barriers that hinder ART adherence and retention in care [28,29]. In the case of Tajikistan, minimal differences in viral suppression rates between urban and rural residents may be due to progress on access to universal healthcare. This is in contrast to the findings of global trends indicating worse HIV outcomes in rural populations [30].

PLHIV residing in Khatlon and Sughd were significantly less likely to achieve viral suppression compared to those in Dushanbe. Key differences in these regions may lie in the development of healthcare infrastructure. Dushanbe, Tajikistan’s capital, has a higher density of healthcare workers, more developed systems of primary care, and access to more laboratory equipment than other regions [31,32]. Consequently, individuals in Dushanbe may benefit from more frequent viral load monitoring, earlier treatment initiation, and greater ART continuity that may result in higher viral suppression levels. In contrast, Khatlon and Sughd regions comprise rural areas with geospatial barriers that hinder timely access to healthcare services [31]. While structural barriers are possible explanations for the observed gaps in viral suppression, another potential driver may be the uneven distribution of key populations and related demographic factors. For instance, a recent study found that the risk of viral non-suppression in Sughd was the highest among both male migrants and nonmigrants [21]. Additionally, Khatlon’s proximity to regional opiate trafficking routes and higher prevalence of injectable drug use may compromise engagement in HIV care and continuity on ART [18]. These intersecting structural and behavioral factors may potentially interact to widen regional disparities in achieving viral suppression.

Interventions to improve viral suppression gaps should prioritize addressing gender and geographic disparities within Tajikistan’s HIV care continuum. Men remain significantly less likely to achieve viral suppression across all thresholds of gender-related measures, possibly highlighting the need for strategies that strengthen their engagement in testing, treatment initiation, and long-term retention in care. Further research is needed to clarify whether these disparities are influenced by gender identity, cultural norms, or systemic inequities within healthcare delivery. Interventions must also target rural populations, who may face greater challenges to care access and retention, suggesting that geographic and structural barriers continue to limit engagement in HIV services. Similarly, in regions such as Sughd and Khatlon, it is possible that structural barriers intersect with a higher prevalence of key populations, underscoring the need for regionally tailored approaches. Future studies are needed to understand viral suppression by behavioral risk among more vulnerable populations, including people who inject drugs (PWID), sex workers, men who have sex with men (MSM), labor migrants, and the incarcerated. In parallel, these studies should implement stricter definitions for viral load thresholds aiming to define viral suppression using U=U initiatives to effectively communicate progress. While our study utilized a robust national registry, limitations include reliance on cross-sectional data, potential underreporting or data quality issues in remote areas, and not supporting causal inference. Additionally, treatment regimens and duration, adherence data, and CD4 count were not used, as the focus for this novel study emphasized treatment outcomes based on demography, utilizing parsimonious model development. However, we acknowledge that these and missing behavioral factors substantially limit our interpretation of viral suppression outcomes, and frame portions of our discussion as speculative hypotheses rather than evidenced conclusions. Future studies could explore these variables and develop more complex statistical models (e.g., test for effect modification of demographics, clinical variables, behavioral factors via interaction terms). We also recognize the geographical limitations in estimating rural versus urban disparities, as certain oblasts in Tajikistan are significantly more rural than others. Despite these limitations, our study provides strong evidence that Tajikistan is moving toward global HIV treatment goals but highlights critical gaps that demand high-quality interventions. Achieving viral suppression across demographics will require continued investment in localized, patient-centered strategies and a commitment to leaving no one behind in the national HIV response.

## Data Availability

The datasets presented in this article are not readily available because they are confidential registry data owned by the Tajikistan Ministry of Health and Social Protection of Population and contain sensitive personal health information. Requests to access the datasets should be directed to Arash Alaei, arash.alaei@csulb.edu, for consideration with the Ministry and relevant ethics committees.

## Abbreviations

The following abbreviations are used in this manuscript:

PLHIV	People living with HIV
ART	Antiretroviral Therapy
U=U	Undetectable = Untransmittable
UNAIDS	Joint United Nations Program on HIV/AIDS
